# Histone Demethylase Modulation: Epigenetic Strategy to Combat Cancer Progression

**DOI:** 10.3390/epigenomes7020010

**Published:** 2023-05-17

**Authors:** Rashmi Srivastava, Rubi Singh, Shaurya Jauhari, Niraj Lodhi, Rakesh Srivastava

**Affiliations:** 1 Department of Zoology, Babasaheb Bhimrao Ambedkar University, Lucknow 226025, Uttar Pradesh, India; 2 Department of Hematology, Bioreference Laboratories, Elmwood Park, NJ 07407, USA; 3 Division of Education, Training, and Assessment, Global Education Center, Infosys Limited, Mysuru 570027, Karnataka, India; 4 Clinical Research (Research and Development Division) Mirna Analytics LLC, Harlem Bio-Space, New York, NY 10027, USA; 5 Molecular Biology and Microbiology, GenTox Research and Development, Lucknow 226001, Uttar Pradesh, India

**Keywords:** epigenetics, transcription, histone demethylases, cancer, histone demethylase inhibitors, cancer epitherapy

## Abstract

Epigenetic modifications are heritable, reversible changes in histones or the DNA that control gene functions, being exogenous to the genomic sequence itself. Human diseases, particularly cancer, are frequently connected to epigenetic dysregulations. One of them is histone methylation, which is a dynamically reversible and synchronously regulated process that orchestrates the three-dimensional epigenome, nuclear processes of transcription, DNA repair, cell cycle, and epigenetic functions, by adding or removing methylation groups to histones. Over the past few years, reversible histone methylation has become recognized as a crucial regulatory mechanism for the epigenome. With the development of numerous medications that target epigenetic regulators, epigenome-targeted therapy has been used in the treatment of malignancies and has shown meaningful therapeutic potential in preclinical and clinical trials. The present review focuses on the recent advances in our knowledge on the role of histone demethylases in tumor development and modulation, in emphasizing molecular mechanisms that control cancer cell progression. Finally, we emphasize current developments in the advent of new molecular inhibitors that target histone demethylases to regulate cancer progression.

## 1. Introduction

Cancer is one of the most complex non-communicable diseases, manifesting in uncontrolled and aberrant cell proliferation that gives rise to cellular aggregates and localized tumors. Approximately 20 million individuals globally are affected by various malignancies, and around 10 million people are dying from them every year [1,2]. Dysregulation of epigenetic changes has also been linked to the development of cellular resistance to therapies and the onset of carcinogenesis [3,4,5,6]. The word “*epigenetics*” describes events that can influence gene expression without modifying the DNA sequence. DNA methylation, histone modifications, and the control of post-transcriptional gene expression by the noncoding RNA are the main mechanisms behind epigenetic regulations [7,8,9,10]. It has been revealed that uncontrolled, dynamic epigenetic modifications can initiate poorly prognosed cancer development. Recent studies suggest fluctuations in the expression of oncogenes and tumor suppressor genes due to uncontrolled epigenetic changes in the malignant cells. This scenario necessitates the exploration of a potential therapy to mitigate cancer incidence [10,11,12] The majority of aberrant, epigenetically modified genes participate in the cell cycle, cellular invasion, DNA repair, and genetic instability pathways, thereby perturbing genomic normalcy [4,5,6,13,14,15,16,17,18,19,20,21,22,23].

Histone modifications are responsible for chromatin compaction, nucleosome dynamics, and transcription regulation [24,25,26]. Dysregulation of these mechanisms, whether by gain or loss of functions, overexpression or suppression, chromosomal translocations, inhibition by promoter hypermethylation, or mutations of the histone-modifying enzymes/complexes, even at the histone modification site, is often observed in the development of cancer [12,27,28]. Depending on the cell type/tissue, variegated histone modifications, resulting in tissue-specific gene expression profiles which characterize certain biological activities at cellular levels, shall establish either normal or disease conditions [9,26,29,30,31,32].

Cellular signals, both internal and external, are subjected to histone modifications. Several chemical modifications occur on histones at various amino acid residues, the most common of which are acetylation, phosphorylation, methylation, and ubiquitylation [6,33,34]. Distinct forms of histone modifications have been found at 130 different residues on the core and linker histones [35]. These histone modifications can be found in the globular core regions of histone proteins, as well as in the amino- or carboxy-terminal tails that extend from the surface of the nucleosome [25]. Among all, histone methylation is imperative in many biological processes, including cell cycle progression, immunological response, and signal transduction [36]. Furthermore, histone methylation/demethylation is associated with diseases such as globin abnormalities and neurological disorders. Histone methylation/demethylation is prominently linked to cellular oncogenesis and proliferation, and it has been found to be altered in many cancer cells [37,38,39].

Because histone methylation is a reversible process, it may be possible to employ this epigenetic regulation to bring about a positive change in the function of oncogenes and tumor suppressor genes in cancers. Considering the tissue-specific functional epigenetic landscape, it is quite tedious to acknowledge the multiple or singular modifications that are consistent in normal cells and therefore also in cancer cells. Hence, it will be equally demanding to extract or invent individual epigenetic modifications, signal transductions, and gene expression profiles that are scaled to a systemic level. In these proceedings, summarizing the exact mechanism of the onset of cancer’s occurrence, therefore leading to treatment options via selections of specific drug targeting and modes of action, is a challenging task per se. There is currently a lot of evidence that suggests the role of unregulated histone methylation in the development of cancer. In the present review, we document a particular subset of *eraser* proteins called histone demethylases, and document their association with the advancement of cancer. We also highlight recently described chemical compounds targeting histone demethylases and their method of action, as well as prospective therapeutic targets. We explore the role of a plethora of demethylases and their inhibitors, which are vulnerable to epigenetic crosstalks, thereby paving the way for epigenetic therapy through drug discovery, targeting, and delivery systems integrated with epi-engineering.

## 2. Insights into Histone Demethylases

Histone methylation is a three-step process that includes the integral roles of “writers”, or histone methyltransferases (HMTs), “readers,” or histone methylation-recognizing proteins, and “erasers,” or histone demethylases (HDMs). Histone methylation and demethylation regulate genes, either by relaxing histone tails to permit transcription factors and other proteins to contact the DNA, or by wrapping histone tails around the DNA, thereby blocking access [40]. These changes impact nucleosomal characteristics and, henceforth, their interactions with other proteins. Histone methylation entails the addition (through writer enzymes) or elimination (via eraser enzymes) of methyl groups, mostly on the lysine (K) or arginine (R) amino acids of histone; however, it has also been witnessed on glutamine, aspartate, and histidine residues [41]. Histone methylation does not affect the molecule’s overall charge, in contrast to acetylation and phosphorylation, wherein the methyl donor in histone methylation processes is S-Adenosylmethionine (SAMe). Lysines can be monomethylated (me1), dimethylated (me2), or trimethylated (me3) on their -amino group, whereas arginines can be monomethylated, symmetrically dimethylated (me2s), or asymmetrically dimethylated (me2a) on their guanidinyl group [41]. Until the discovery of lysine-specific demethylase 1 (LSD1), which demethylates mono- and dimethyl groups in H3K4 [42], it was thought that methylation of histone residues was permanent, hereditary, and irreversible. The dynamics of histone methylation and demethylation on gene regulation are now better understood due to the ground-breaking discovery of histone demethylases in 2004 [42]. Histone demethylases can mainly be divided into two groups, based on their functions when demethylating histones. The first class of histone demethylases (LSD1, as aforementioned) belongs to the family of enzymes known as flavin-dependent amine oxidases. The second class of histone demethylases belongs to the family of JmjC domain, which catalyzes the oxidation of ferrous ions and uses ketoglutarate as a cofactor to demethylate histone lysine [11]. Additionally, the cohort of histone lysine demethylase (KDM) is classified into sub-families KDM1 to KMD9, and other types of proteins that are also involved in histone demethylation [43] (Table 1).

LSD1 has a flavin-dependent amine oxidase (AO) domain and a SWIRM domain. With the help of the AO domain, it oxidizes the amine in a FAD-dependent way to remove H3K4me1/2, while the SWIRM domain identifies and binds to DNA [44]. The zinc-finger domain, in addition to the SWIRM and AO domains, is present in LSD2, a paralog of LSD1; while LSD2 demethylates gene body regions, LSD1 demethylates the promoter and enhancer regions of genes [45]. The catalytic JmjC domain is a characteristic feature of the second family of KDM, which can be categorized into seven subfamilies in humans based on the homology of the JmjC domain. Two cofactors, Fe (II) and 2-oxoglutarate, are bound in the JmjC domain of the enzyme, and function as cofactors in the catalytic process to create a highly active oxoferryl (Fe (IV) = O) intermediate that hydroxylates the -methyl groups of the substrate methylated lysine. The resultant lysyl hemiaminal is unstable and disintegrates, releasing the nitrogen’s methyl group as formaldehyde. JmjC demethylase members have been revealed to demethylate the trimethylated lysines, demonstrating that this mechanism is capable of demethylating lysine in all three methylation states. (mono-, di-, and tri-methylated lysine) [11,27,43,46,47].

The human genome codes for five protein arginine deiminases (PADs), which function to remove methyl groups from arginine. These enzymes transform peptidyl arginine into citrulline in a calcium-dependent manner. It has been determined that PAD4 is a demethylase that transforms monomethylated arginine into citrulline by demethylating histones [47,48]; however, whether PAD4 performs as a strict histone demethylase is subject to discussion. JMJD6, a member of the Jumonji-domain histone demethylase (JHDM) family of histone lysine demethylases, is shown to have histone arginine demethylase activity rather than lysine demethylase activity [49]. Additionally, the Jumanji C domain-containing subset of lysine demethylases KDM3A, KDM4E, KDM5C, and KDM6B also exhibits a site-specific arginine demethylase function [50].

## 3. Histone Demethylase Dysregulation and Cancer

**Figure 1:**
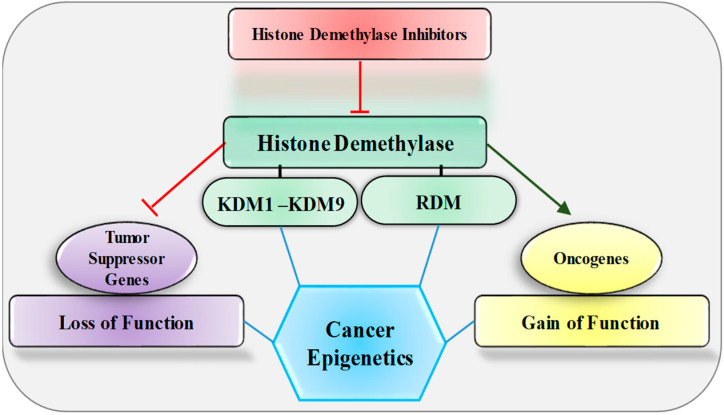
Effect of histone demethylase and its inhibitors on oncogene and tumor suppressor genes. KDM, lysine-specific histone demethylase; RDM, arginine-specific histone demethylase.

Histone demethylases are engaged during transcription regulation in many human diseases including cancer; therefore, many research groups target them for therapeutic intervention [51,52]. Dysregulation of demethylases in different cancer types affects the transcription regulation of oncogenes and tumor suppressor genes (Figure 1). The potential roles of histone demethylases in cancer show subtle variations in the method of action, largely oncogenic, with the exception of some demonstrating tumor suppressor function too. For instance, LSD1 leads to tumor formation due to its capacity to silence tumor suppressor genes as a transcriptional co-repressor, mainly through H3K4 demethylation [53]. In cancer cells, histone demethylases contribute to tumor progression by removing suppression marks from oncogenes and active marks from tumor suppressor genes. However, this can be corrected by inhibition of histone demethylase, with significant potential for the regulation of gene expression through treatment with small molecules. Accumulating evidence suggests that the epigenetic regulatory functions of histone demethylases are now complex (and challenging to understand) in their regulation of the expression of oncogenes and tumor suppressor genes in different cancer types. Demethylase dysregulation in tumors can have various effects based on the source of tissue, the existence of certain other mutations, and the involvement of other gene expression networks, due to the interaction of combinatorial histone modifications, with additional regulatory processes influencing the overall biological pathways. Gene mutations or translocations in the histone demethylase family are uncommon, while variations in expression levels for these family members are more prevalent [54]. Thus, examining their underlying mechanism of action in a tumor environment, along with their inhibition within a tumor, may resolve the development of successful cancer therapeutics. Histone demethylase dysregulation, especially in cancers and the variety of regulatory abnormalities seen therein as implications, suggests that a suitable balance between histone methylation and demethylation is necessary for cellular homeostasis.

**Figure 2:**
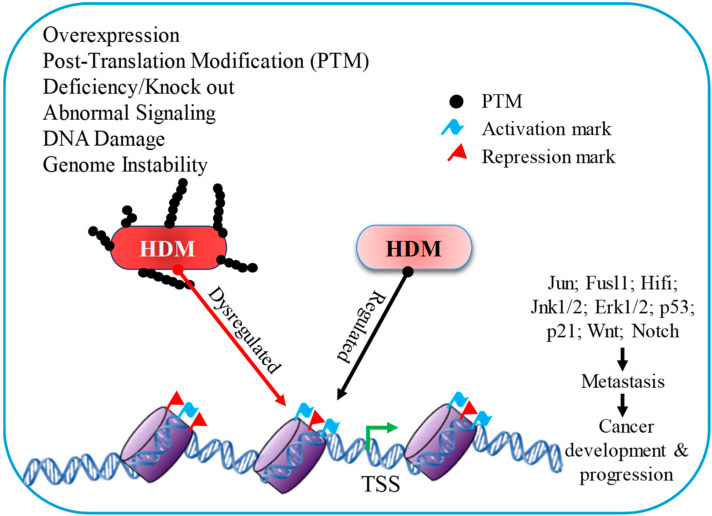
Histone demethylase has a distinct influence on gene expression due to diverse signaling mechanisms throughout cancer development and progression.

Previous studies have discovered a significant link between cancer and histone demethylases, with multiple instances of histone demethylase overexpression in tumor cells (Figure 2). One possible explanation for demethylase upregulation in cancer cells is hypoxia. Hypoxia is a widespread condition in tumor tissues, because their blood vessel networks are frequently insufficient to feed starving cells with the necessary quantity of oxygen. Yang et al. (2009) revealed that this deprivation of oxygen induces the Jumonji family of KDM to a hypoxia-inducible factor (HIF)-dependent mechanism, which is active in hypoxic conditions and helps in increasing the expression of Jumonji family genes [55]. In vivo, demethylase activity is hardly affected by low oxygen concentrations, making histone demethylases a viable marker for a cancer cell’s biological response to hypoxia.

Understandably, the removal of active marks (H3K4, or H3K36) from tumor suppressor genes or the removal of repressive marks (H3K9, or H3K27) from oncogenes in cancer cells could well be related to the overexpression of demethylases. This is the primary rationale for designing therapies through modulating demethylase activity. Overexpression of *KDM1* in tumor cells promotes tumor growth by silencing tumor suppressor genes via the demethylation of dimethylated H3K4 [56]. Related studies also show that overexpression of demethylase *KDM1* contributes to human carcinogenesis through chromatin regulation in various cancers [56]. For instance, *KDM1* is overexpressed in bladder carcinomas but exhibits significantly decreased proliferation when treated with small interfering RNA (siRNA) matching to the *KDM1* gene. The overexpression and carcinogenic activities of *KDM1* have been also reported in breast, colorectal, liver, lung, hypopharynx, and prostate, cancers [56,57,58,59,60,61,62]. This renders key candidature to LSD1/KDM1 as a possible cancer therapeutic. Other groups of KDM have different expression patterns in diverse cancers, a composite understanding of which is essential to clarify their intricate functionalities in cancer cells. *KDM2A* overexpression also increases lung cancer growth by means of epigenetically increasing ERK1/2 and JNK1/2 signaling. In lung cancer, the dual-specificity protein phosphatase 3 (*DUSP3*) expression is suppressed by KDM2A-dependent H3K36 demethylation at its promoter, which raises ERK1/2 and JNK1/2 functions [63]. Observably, *KDM3* overexpression promotes tumor growth in a variety of cancers, including prostate, breast, colon, lung, bladder, neuroblastoma pancreatic, ovarian, and multiple myeloma [64].

Overexpression of *KMD3C/JMJD1C* promotes in vivo cell proliferation and tumorigenicity in a demethylase-independent manner, enhancing glycolytic and oxidative enzymes, which maintains leukemic cell bioenergetics and leads to severe acute myeloid leukemia (AML) characteristics [65]. *KDM3* is overexpressed in a variety of cancers, in which it aids in tumor development. KDM3A/B substantially regulates the expression of Wnt or beta-catenin target genes *c-Myc*, *MMP9* (matrix metallopeptidase 9), and *cyclin D1* in colorectal cancer, thereby promoting the ability of colorectal cancer stem cells to undergo self-renewal [66,67,68]. Similarly, histone demethylase *KDM4B/JMJD2B* is overexpressed in gastric cancer and is a requisite for tumor cell proliferation. Knockdown of *KDM4B* strongly affects clonogenicity; the growth of xenograft tumors in mice is reduced, apoptosis is induced, and *p53* and *p21(CIP1)* expression is accelerated [69].

*KDM5*, which demethylates H3K4me2/3, has been reported to be overexpressed in human breast, head and neck, gastric, prostate, and bladder cancers [70]. *KDM5B* overexpression in hepatocellular carcinoma resulted in an undesirable prognosis [71]. The expression of *E2F1* and *E2F2* seems to be transcriptionally suppressed by KDM5B knockdown, which inhibits cell cycle progression from the G1 to S phase and the development of cancer cells [71]. KDM5D downregulation has been linked to a poor survival rate in colorectal cancer. *KDM5D* overexpression effectively suppressed colorectal cancer development and metastasis in vitro and in vivo. Additionally, KDM5D inhibited tumor growth in colorectal cancer by demethylating E2F1 and suppressing FKBP4 transcription, thereby suggesting that it could possibly serve in the management of colorectal cancer in males [72]. KDM6B has been reported to be downregulated in neuroblastoma stem-like cells and to have tumor-suppressive functions. Its overexpression reduces proliferation while increasing genes for differentiation, indicating the control of neuroblastoma cell differentiation by a KDM6B demethylase activity-dependent epigenetic mechanism [73]. Multiple myeloma cells have high levels of *KDM6B* expression, and when *KDM6B* was knocked down, the growth of these cells and their survival were limited [74]. It is well known that transforming growth factors are modulated by *KDM6B* overexpression to increase the motility and invasion of ovarian cancer cells [75,76]. In the KDM7 family, KDM7A and KDM7B promote the formation of cancer; interestingly, KDM7C/PHF2 inhibits tumor development [77]. *PHF8/KDM7B* overexpression has been shown to act as an oncoprotein in a variety of cancers, including, hepatocellular carcinoma, laryngeal and hypopharyngeal squamous cell carcinoma, and non-small cell lung cancer [78,79,80]. The tumor-promoting function of PHF8 is triggered by the overexpression of oncogenic *miR-21*, which suppresses the tumor suppressor gene *PTEN*, leading to tumor growth and invasion in non-small cell lung cancer [78,81]. KDM8/JMJD5 protein, the newest KDM family member, performs several crucial biological functions. The increase in the *cyclin A* level, regulation of the *p53* and *p21* expression, and the interaction with spindle microtubules are all molecular mechanisms orchestrated by KDM8 within the cell cycle [11]. *KDM8* overexpression has been linked to colon and breast cancer in several investigations, establishing that *KDM8* deletion impairs cancer cell growth [70,82].

JMJD6 is involved in the arginine demethylation of H3R2me1/2 and H4R3me1/2, and has also been found to be significantly expressed in a variety of cancerous tumors [83,84]. Not only is JMJD7 upregulated in head and neck squamous cells [85], its knockdown or a defective, enzymatically inactive variant of JMJD7 in breast cancer cells significantly reduces cell growth [86]. JMJD1B/KDM3B is also involved in the arginine demethylation of H4R3me2s and their intermediate, H4R3me1 [87]. The loss of KDM3B disrupts an epigenetic program by inhibiting p53-p21 signaling, and enhancing cancer cell development and survival [87]. Histone demethylases may not always overexpress; in fact, they may even be downregulated in some malignancies [88,89,90,91]. However, this depends more on the lysine/arginine residues of oncogene and tumor suppressor genes (Figure 2).

## 4. Histone Demethylase Inhibitors: Sentinel of Cancer

Our knowledge on the role of epigenetics in tumorigenesis and carcinogenesis has greatly benefited from the recent discovery of histone demethylases and their role in the regulation of post-translational chromatin modifications, which may provide new therapeutic means of combatting cancer. The effect of histone demethylases on lysine and arginine residues, along with their potential therapeutic value of targeting these enzymes with the help of histone demethylase inhibitors, is a crucial value addition to the treatment of cancer [92]. Over the past few years, there has been a sharp surge in interest in identifying KDM inhibitors and understanding their inhibitory mechanisms (Table 2) [93,94,95,96]. Small compounds and altered peptides are examples of known KDMs inhibitors. In vitro and in vivo assessments have been carried out on a number of these inhibitors to determine their physiological and pathological effects. In particular, eight KDMs inhibitors are now in the clinical stages for their prospective applicability in antineoplastic treatment; the doors are still open for future histone demethylase inhibitor developments [64,97,98,99,100]. According to the targeting mechanism, these new KDM inhibitors are divided into two groups: first, those that inhibit JmjC KDMs, and second, those that inhibit FAD-dependent KDMs [64,97,101]. Based on their enzymatic processes, KDM inhibitors may be categorized into four classes: substrate- and cofactor-independent inhibitors, metal-cofactor disruptors, competitive substrate inhibitors, and -KG/2-OG cofactor mimics, respectively.

Several LSD1 inhibitors, including the group of CBB1003 TCP (Tranylcypromine), ORY-1001, GSK-2879552, IMG-7289, INCB059872, CC-90011, ORY-2001, and MC3324, suppress the growth of colorectal cancer cells AML, acute lymphoblastic leukemia, solid tumors, and breast cancer [67,100,102,103,104,105,106]. Identification of the 3D structure is necessary for understanding the molecular mechanism behind LSD1’s function, and this knowledge is paving the way for the creation of new inhibitors with medicinal value. LSD1 may possibly serve as a marker for the early detection and management of malignant tumors [107,108]. LSD2/AOF1/KDM1B is a different member of this amine oxidase protein family, and although structurally comparable to LSD1, LSD2 does not participate in the complexes that represent chromatin [45]. LSD2’s linkage to the control of the inflammatory process through NF-kB and its dependence on the mono- and di-methylation of histone H3K4 are crucial for the production of induced pluripotent stem cells, and are also connected to genomic imprints [109,110].

Many LSD1 inhibitors have been trialed thus far. The monoamine oxidases MAO-A and MAO-B are also inhibited by the LSD1 inhibitors pargyline and tranylcypromine (TCP), which have been successfully utilized in the symptomatic treatment of depression. Phase I and II clinical studies for LSD1 inhibitors against cancer are now being conducted with iadademstat (ORY-1001) and GSK2879552. While GSK2879552 is being evaluated for the treatment of AML (NCT02177812) and small cell lung cancer, ORY-1001 is being tested for the treatment of relapsed or refractory acute leukemia. Additionally, GSK2879552 is being tested in people with high-risk myelodysplastic syndrome, both independently and in conjunction with the DNA methyltransferase inhibitor azacitidine (NCT02929498). Recent studies indicate that LSD1 may have a role in resistance to trans-retinoic acid; comparable trials are being undertaken in patients with relapsed and/or refractory solid tumors, and non-Hodgkin’s lymphoma in conjunction with tranylcypromine (TCP+ATRA; NCT02261779, NCT02273102) and CC-90011 (NCT02875223) [100,111,112]. The first trivalent rhodium-based LSD1 inhibitor was described by Yang et al., and showed selectivity over other related enzymes such as KDM2b, KDM7, and monoamine oxidase [113,114]. The LSD1-H3K4me2 connection was broken down by this metal complex in human prostate cancer cells, which improved the amplification of the *p21*, *FOXA2*, and *BMP2* gene promoters [101,115,116,117,118]. KDM2B knockdown or use of GSK-J4 inhibitors lowered the frequency, differentiation ability, and survival of glioblastoma stem-like cells, the inhibition of the DNA repair capacity of GBM cells, and chemoresistance [119].

The KDM3 subfamily primarily consists of four proteins (KDM3A−D), all containing the catalytic Jumonji C domain (JmjC) at their C- terminus. The expression and deregulation of *KDM3* have been increasingly found to be linked to the development of many malignancies [64,120]. In fact, a poor prognosis, chemo-resistance, and patient recurrence are all implied by substantial histone methylation alterations [121]. KDM3 proteins, especially KDM3A, have the potential to function as oncoproteins, making them prospective therapeutic targets for the treatment of cancer. Only a few KDM3 inhibitors have been discovered so far, yet they are pan-KDM inhibitors and not particularly selective for the KDM3 subfamily; however, the development of selective KDM3 inhibitors is critical, and in this context, a few KDM3 inhibitors that bind to KDM3’s JmjC domain have been described [67]. A carboxamide-substituted benzhydryl amine, CBA-1, functions as a KDM3A/3B inhibitor (typically inhibiting KDM3A), causes increased levels of H3K9me2, inhibits Wnt targets (Axin2, c-Myc, and Cyclin B1), and prevents colorectal cancer cell proliferation [122]. A JmjC domain inhibitor-16, JDI-16, binds and displays a moderate affinity to KDM3C and KDM3B, demonstrating vigorous action against malignant hematopoietic cells [123].

KDM4 inhibitors are categorized into multiple types based on functionality, including 2-OG cofactor mimics, metal cofactor disruptors, histone substrate-competitive inhibitors, natural inhibitors, and peptide inhibitors [98,124,125,126]. JIB-04, the most advanced preclinical KDM4 inhibitor, inhibits growth and lowers tumor burden in non-small cell lung cancer and breast cancer, both in vitro and animals, and targets KDM4A, KDM4B, and KDM4E [127,128]. Additionally, JIB-04 treatment decreased colony formation, development, and migration in vitro, and decreased tumorigenic activity in a colorectal cancer model in vivo. Downregulation of Wnt signaling pathway genes, which are crucial for promoting carcinogenesis, has been suggested to be the cause [129,130]. ML324 is a different KDM4 inhibitor in the arsenal; it specifically targeted KDM4B and KDM4E, suppressed prostate cancer proliferation both in vitro and in vivo, and decreased tumor volume and growth in a triple-negative breast cancer mice model [131,132,133,134,135]. Ciclopirox, a small-molecule antifungal drug, has been identified as a pan-histone demethylase inhibitor. It inhibits many histone demethylases, including KDM4B, which is involved in MYC function [113]. Ciclopirox has been identified to be a powerful KDM4B inhibitor that diminishes the growth of neuroblastoma cells while having minimal impact on healthy nerve cells, suggesting that it could be employed to counter neuroblastoma [113]. KDM4/KDM4A inhibitors such as NCDM-32B, JmjN peptide, Purpurogallin (9bf), PKF118–310, and ML324 cause inhibition of tumor cell proliferation in breast cancer, and showcase antiproliferative activity in prostate cancer [136,137,138,139,140]. The inhibitors from the Jumonji domain-containing protein family of KDMs, such as JIB-04, target colorectal cancer stem cell (CSC) growth, fight against colorectal cancer, and reduce cancer growth in lung and prostate cancer cell lines [70,140]. JMJD2C inhibitor- IOX1 is used to reduce the proliferation and migration of vascular smooth muscle cells and breast cancer [134,141,142]. B3 compound, also known as NCGC00244536, is particularly employed as a KDM4B inhibitor [143]. It binds directly to the demethylase catalytic site of the KDM4 protein, reducing its activity in addition to suppressing the development of many cancer cells, while having minimal toxicity to and adverse effects on normal tissue cells [143,144]. Furthermore, it prevented KDM4B from binding to the polo-like kinase-1 (PLK1) promoter, showing a possible mechanistic treatment approach to prostate cancer and tumors expressing high levels of *KDM4B/PLK1* [143].

The KDM5 subfamily catalyzes H3K4me2/3 marks, in contrast to other KDM sub-families that target several lysine residues [145,146]. As a result, KDM5A-D enzymes may contribute to the downregulation of tumor suppressors and oncogenes [147]. There is a mounting body of evidence to suggest that KDM5 dysregulation is detrimental to numerous cancer types. PBIT, an H3K4me3 demethylase inhibitor, also makes cancer cells more sensitive to radiation than the H3K27me3 demethylase inhibitor [148]. KDM5A-mediated H3K4me3 demethylation inhibits the expression of the *p16* and *p27* tumor suppressor genes in breast cancer [114]. By suppressing the genes that are targets of the tumor suppressors NOTCH1 and NOTCH2, KDM5A also encourages the growth of small cell lung cancer cells [149]. Myeloma cell growth is halted by powerful and specific KDM5 inhibitors, verifying the oncogenic functions of KDM5A. The KDM5A inhibitor CPI-455 lowers the quantity of lung cancer cells that are resistant to the EGFR inhibitor, erlotinib, and the number of melanoma cells that are resistant to the B-Raf inhibitor, vemurafenib, by inhibiting H3K4 demethylase activity. KDM5 inhibition prevents MM1S myeloma cell growth and cellular demethylation of H3K4me3 at the transcription start sites [116], while another, Quercetin (WO2007104314), causes inhibition of esophageal carcinoma cell lines and osteosarcoma [124].

Another sophisticated KDM5 inhibitor is EPT-103182, a small molecule drug that targets KDM5B, which has been demonstrated to reduce tumor development in xenograft models in a dose-dependent manner, and to have an anti-proliferative impact in hematological and solid cancer cell lines [150,151]. An additional KDM5 inhibitor, PBIT, has been found to specifically target and inhibit KDM5B [95]. PBIT therapy has been found to reduce the growth of breast cancer by upregulating and de-repressing the tumor suppressor, HEXIMI, in vitro [152]. 

KDOAM-25, a KDM5 enzyme inhibitor, has biochemical half maximum inhibitory concentration values for KDM5A-D in vitro, a high degree of selectivity for other 2-OG oxygenases subfamilies, and no off-target action on a panel of 55 receptors and enzymes. KDOAM-25 has a half maximum effective concentration and strong selectivity against other demethylases in human cell assay methods, in addition to being overexpressed in multiple myeloma, and is associated with worse overall survival. KDOAM-25 treatment of multiple myeloma MM1S cells results in an enhanced global H3K4 methylation at transcriptional start sites besides decreased proliferation [116]. JQKD82 dihydrochloride is a cell-permeable, selective inhibitor of KDM5, with little action against other KDMs. It preferentially binds KDM5A over other KDM5 isoforms, promotes H3K4me3 hypermethylation, and assists in MYC target downregulation and RNAPII phosphorylation; furthermore, it inhibits multiple myeloma cells [74].

*KDM6B* has been linked to poor survival and has been observed to be overexpressed in diffuse large B-cell lymphoma (non-Hodgkin lymphoma). Moreover, to inhibit *KDM6B* expression in B-cell lymphoma cells, a small molecule KDM6 inhibitor, GSK-J4, has been found to make the lymphoma cells more susceptible to chemotherapeutic drugs [153]. GSK-J4, which targets KDM6A and KDM6B, suppresses the production of pro-inflammatory cytokines through human macrophages, albeit its specificity is questionable [93,101,154]. Apparently, GSK-J4 therapy limits cell growth and induces cell cycle arrest and apoptosis in primary human T-cell acute lymphoblastic leukemia lines by inhibiting KDM6B activity [155]. Targeting KDM6B with GSK-J4 suppresses cell proliferation and colony formation in AML cell lines, as well as tumor development in an AML xenograft mice model by enriching the H3K27me3 repressive mark in HOX genes’ transcription start sites [103]. GSK-J4 therapy also stifled the proliferation of castration-resistant prostate cancer cells by decreasing androgen receptor-mediated transcription, in addition to inhibiting glioma cell proliferation in vitro in a dose-dependent manner [96,156]. In NSCLC cell lines, GSK-J4 in conjunction with the anti-diabetic medicine metformin promoted cell death and decreased cellular growth [101,157,158], while another study indicated that GSK-J4 treatment sensitized diffuse large B-lymphoma cells to chemotherapy treatments [153]. In neuroblastoma cell lines, GSK-J4 reduced cell proliferation, increased apoptotic markers, and brought down tumor development in an in vivo model of the disease [159]. UTX/KDM6A inhibitors such as MC3324 arrest growth and induce apoptosis in hormone-responsive breast cancer, emulating tumor suppression [104,160]. A JumonjiC inhibitor, JIB-04, could be used as a potential therapeutic for targeting taxane-platin, chemo-resistant NSCLCs [161,162].

KDM7A is another member of the JmjC family, which includes PHF2 (KDM7C) and PHF8 (KDM7B) [163]. A study has reported the discovery of a cell-permeable KDM2A/7A inhibitor that exhibits more than or equal to 75-fold selectivity relative to other JmjC KDM sub-families [164]. In 2012, Wagner et al. identified amiodarone, an antiarrhythmic drug, as an inhibitor of KDM5A (PHD3). Later, inhibition of the binding and catalytic activities of KDM7A/B (PHD-JmjC) by derivatives of amiodarone was established. In addition, the PHD fingers of KDM7s were inhibited by WAG-003 and WAG-005 to varying degrees of potency, with WAG-005 being the most impressive [165,166].

EZH2 inhibitors (GSK343 and GSK126) and KDM8 knockdown both re-sensitize the cells toward enzalutamide. In the cytosol, KDM8 associates with PKM2, the gatekeeper of pyruvate flux, and translocates it into the nucleus, where the KDM8/PKM2 complex serves as a coactivator of HIF-1α to upregulate glycolytic genes [70]. Inhibitors of KDM8 have been under study and have been upon the verge of clinical correlations; still, the mechanism involved is quite complex. Some studies reveal that KDM8 knockdown inhibited cell growth, which can be utilized as a potential check for tumorigenesis or oncogenesis [37,167,168,169].

It is surprising to note that SKLB325, which inhibits the activity of arginine demethylases, JMJD6, has been found to suppress cell proliferation, induce apoptosis, enable antitumor activities, and display effectiveness in the treatment of ovarian cancer [49,170]. The JMJD6 inhibitor SKLB325 has shown potent anti-ovarian cancer effects in an intraperitoneal xenograft model [170]. The compound WL12 has been shown to inhibit JMJD6 enzymatic activity, as well as the JMJD6-dependent proliferation of cervical and liver cancer cells [171].

Natural bioactive products are one of the most abundant sources of emerging biological compounds, and have been utilized for many years to treat a variety of diseases including cancer [172,173,174]. Furthermore, it has been discovered that natural substances such as cyclic peptides, flavonoids, alkaloids, and diarylheptanoids act as HDM inhibitors to restrain tumor growth and metastasis [77,92,101]. Arborinine, a naturally occurring compound produced by *Glycosmis parva*, is a reversible LSD1/KDM1 inhibitor that is capable of reducing adriamycin-resistant gastric cancer cell growth by boosting H3K4me1 and H3K9me1/2 levels [175]. A plant growth regulator, daminozide (N-(dimethylamino)succinamic acid) specifically inhibits KDM2 and KDM7 [176]. Daminozide’s inhibition of KDM2A causes a decrease in the stemness and chemoresistance of breast cancer cells [177]. Purpurogallin, a naturally occurring phenol derived from oak nutgalls, has been revealed to have antioxidant, anticancer, and anti-inflammatory properties. It was discovered that the purpurogallin molecule and its derivatives are natural KDM4A inhibitors, which triggered the search for an intriguing therapeutic anticancer drug [139]. Silibinin, the active ingredient in the medicinal plant milk thistle, appears to inhibit JMJD5/KDM8 in cases of oral squamous cell carcinoma [178]. Recently, databases such as COCONUT, DrugBank, and FDA have been screened for potential KDM4 family inhibitors of natural origin, based on molecular docking and molecular dynamics approaches. These candidates may facilitate multitarget therapies in cancer [174]. The most important KDM4 inhibitor is caffeic acid, a naturally occurring substance found in several sources, including *Eucalyptus globulus*, which has been discovered to primarily target KDM4C and has strong anti-cancer efficacy against esophageal cancer, both in vitro and in vivo. Myricetin is another natural product, and a potential KDM4 inhibitor [179,180]. Contemporarily, esophageal cancer patients are being considered for clinical research for testing caffeic acid, which is capable of inhibiting KDM4C’s demethylation activity [94,181]. Curcuminoids, derivatives of curcumin, are favorable in reducing JMJD2C/KDM4C activity in vivo, and could be deployed in the treatment of colon cancer [182].

## 5. Perspective and Conclusions

In summary, it can be concluded that histone demethylase inhibitors actively participate as therapeutic targets in a wide range of cancer types, including prostate cancer, breast cancer, colon cancer, lung cancer, liver cancer, bladder cancer, neuroblastoma, pancreatic cancer, ovarian cancer, and multiple myeloma, and new findings from ongoing research will probably add to this list. Histone lysine methylation and demethylation are important mechanisms for regulating chromatin via its relaxation or condensation, and transcriptional activation or inactivation; these processes directly correlate to the gene expressions of the cancer progression pathways. The epigenetic control of gene expression orchestrated by histone methylation is extremely complex due to many lysine residues, and some of the arginine residues can be methylated into distinct states. This control of expression robustly corresponds to several interdependent pathway complexes, yet new findings confirm that this complex mechanism is very definitive, and bears the potential to develop epigenetics-based therapies. For instance, several inhibitors of the KDM4 family proteins are being screened, tested, and developed, relative to the vitality of the KDM4 family as an effective target; impeding the KDM4 family affects malicious protein interactions in the cancer cells [174,183]. Notwithstanding, powered by discoveries from various research groups thus far, the role of histone methylation events in the development of cancer is now well recognized. HDMs are particularly important targets in epigenetic drug development pipelines because of their drug competency. While great progress has been made in defining signaling pathways in many malignancies, and developing high-quality chemical probes and inhibitors for some subfamilies, such as KDM1, KDM2, KDM3, KDM4, and KDM5, much remains unclear about others, such as KDM8 and KDM9. Overexpression of some specific KDM subfamily members, such as KDM4, promotes cancer cell proliferation, invasion, migration, DNA damage, tumor angiogenesis, and metastasis. Blocking the activity of the KDM4 enzyme renders them druggable targets with therapeutic effects; several KDM4 inhibitors have already been identified as anticancer drugs in vitro [184].

In a cancerous tumor, the expression of cell regulatory genes affects transcriptional or translational levels, and deregulation eventually enhances the progression of cancer cells. There is still apprehension surrounding the number of genes whose expression is perturbed when they deregulate within the tumor, and how the crosstalk occurs between them. Yet, we do know that they indirectly affect each other’s cellular functions for the benefit of cancer cell growth, and work synergistically in the progression of cancer. HDMs interact among each other and with several other proteins to maintain cellular functions and genomic stability, and may lead to DNA lesions and dysregulation-related activity if a cell fails to maintain this balance. Placing HDM at the center, we have recapped our knowledge based on state-of-the-art research and possible discourses of major regulatory genes in cancer cells. Further, with corroboration from in silico studies, and harnessing the potential roles of natural products via epigenetic regulatory mechanisms of histone demethylase activity, improvised drug design protocols and delivery systems for the prevention of cancers may be delivered. We anticipate that the control of dysregulated functions and the interruption of the synergistic network in cancer cells can be scaled up to a therapeutic level, and a combination of HDM inhibitors may effectively tackle malignant cells.

## Figures and Tables

**Table 1:** List and site of lysine- and arginine-specific histone demethylase.

Family	Coding Gene	Other Names	Site of Histone Modification
KDM1	KDM1A	LSD1	H3K4me1/2, H3K9me1/2
	KDM1B	LSD2	H3K4me1/2, H3K9me1/2
KDM2	KDM2A	JHDM1A	H3K36me1/2
	KDM2B	JHDM1B	H3K4me3, H3K36me1/2, H3K79me2/3,
KDM3	KDM3A	JMJD1A	H3K9me1/2
	KDM3B	JMJD1B	H3K9me1/2; H4R3me2/me1
	KDM3C	JMJD1C	H3K9me1/2
	KDM3D	HR, ALUNC	H3K9me1/2
KDM4	KDM4A	JMJD2A	H3K9me3, H3K36me3, H4K20me3
	KDM4B	JMJD2B	H3K9me2/3, H3K36me2/3
	KDM4C	JMJD2C	H3K9me2/3, H3K36me2/3
	KDM4D	JMJD2D	H3K9me2/3, H3K36me2/3,
	KDM4E	JMJD2E	H3K9me2/3, H3K36me2/3
KDM5	KDM5A	JARID1A	H3K4me2/3
	KDM5B	JARID1B	H3K4me2/3
	KDM5C	JARID1C	H3K4me2/3
	KDM5D	JARID1D	H3K4me2/3
KDM6	KDM6A	UTX	H3K27me2/3
	KDM6B	JMJD3	H3K27me2/3
	KDM6C	UTY	H3K27me3
KDM7	KDM7A	JHDM1D	H3K9me1/2, H3K27me1/2, H4K20me1/2
	KDM7B	PHF8	H3K4me3, H3K9me1/2, H3K27me1/2, H3K36me2, H4K20me1/2
	KDM7C	PHF2	H3K9me1/2, H3K27me1/2, H4K20me3
KDM8	KDM8	JMJD5	H3K36me2/3, H3R2me1/2, H4R3me1/2
KDM9	KDM9	RSBN1	H4K20me2
Other types	JMJD6	PSR	H3R2me1/2, H4R3me1/2
	JMJD7	PLAEG4B	H3Rme1/2, H4Rme1/2
	JMJD9	RIOX1	H3K4me1/2/3, H3K36me2/3
	JMJD10	RIOX2	H3K9me3
	JARID2	JMJ	H3K9me1, H3K27me3

**Table 2:** Usage of histone demethylase inhibitors in different types of cancers.

Family	Inhibitors	Inhibitory Mechanism in Cancer
KDM1	CBB1003; TCP; ORY-1001, GSK-2879552, IMG-7289, INCB059872; CC-90011; ORY-2001; MC3324, 4SC202; SP-2577; CC-90011; Arborinine	Colorectal cancer; acute myeloid leukemia; acute lymphoblastic leukemia; solid tumors; breast cancer
KDM2	(S,S)-6; Daminozide, GSK-J4;	Cervical cancer; glioblastoma cell
KDM3	IOX1, CBA-1, JD1-16	Colon cancer; leukemia cell lines; hematopoietic malignant
KDM4	NCDM-32B; Purpurogallin (9bf); IOX1; Curcuminoids; Caffeic acid; JIB04; ML324, QC6352; TACH101; SD70	Breast cancer; prostate cancer; colon cancer; non-small cell lung cancer; xenograft models of breast and colon cancers; colorectal cancers; esophageal squamous cell cancer; glioma xenograft tumors
KDM5	Quercetin, EPT103182; PBIT; KDOAM-25; CPI-455; KDM5-inh1	Oesophageal carcinoma cell lines; osteosarcoma; oral squamous cell carcinomas; glioblastoma cells; cervical cancer; breast cancer; multiple myeloma cells; ovarian cancer
KDM6	GSK-J1; GSK-J4; MC3324; Caffeic acid	Squamous cell carcinoma; breast cancer; glioma leukemia; mice xenograft models;
KDM7	Daminozide, (S,S)-6	Cervical cancer
JMJD6	SKLB325; 7p	Ovarian cancer
